# Supplementary material for: Mutation-profile-based methods for understanding selection forces in cancer somatic mutations: a comparative analysis
Source: Oncotarget. 2017 Jul 19;8(35):58835–46. doi: 10.18632/oncotarget.19371 (PMC5601697; doi:10.18632/oncotarget.19371)
Supplement: Supplementary file 1 [file oncotarget-08-58835-s001.pdf]

Mutation-profile-based methods for understanding selection forces in cancer somatic mutations: a comparative analysis

SUPPLEMENTARY MATERIALS

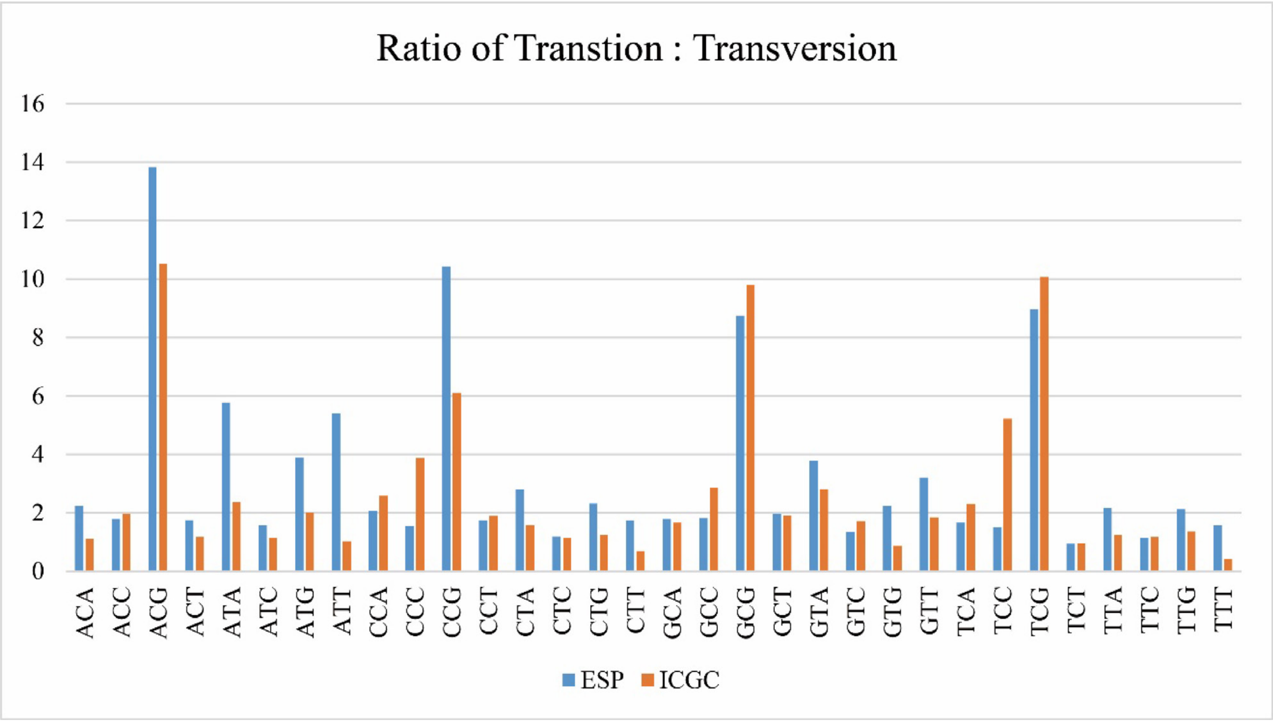

Supplementary Figure 1: The ratio of transition to transversion for each trinucleotide context, according to the exonic mutation rate profiles of ESP and ICGC.

Supplementary Table 1: Selective pressures among species, populations and cancer cells for 16,953 human genes. See Supplementary_Table_1

Supplementary Table 2: List of cancer positively and negatively selected genes ($\chi 2$ test, $p < 10^{-5}$). See Supplementary_Table_2

Supplementary Table 3: Functional enrichment analysis of cancer positively and negatively selected genes ($p < 0.01$, FDR < 0.1). See Supplementary_Table_3

Supplementary Table 4: Comparison of gene expression for 47 negatively selected genes between tumor and normal tissues. See Supplementary_Table_4

Supplementary Table 5: List of cancer positively and negatively selected genes in 20 cancer types ($\chi 2$ test, $p < 0.01$). See Supplementary_Table_5
